# The Effects of Progressive Resistance Exercise on Recovery Rate of Bone and Muscle in a Rodent Model of Hindlimb Suspension

**DOI:** 10.3389/fphys.2018.01085

**Published:** 2018-08-13

**Authors:** Hansol Song, Suhan Cho, Ho-Young Lee, Hojun Lee, Wook Song

**Affiliations:** ^1^Health and Exercise Science Laboratory, Institute of Sport Science, Seoul National University, Seoul, South Korea; ^2^Department of Nuclear Medicine, Seoul National University Bundang Hospital, Seongnam, South Korea; ^3^Department of Rehabilitation Medicine, Seoul National University Bundang Hospital, Seongnam, South Korea; ^4^Department of Sports and Health Science, Kyungsung University, Busan, South Korea; ^5^Institute on Aging, Seoul National University, Seoul, South Korea

**Keywords:** skeletal muscle, bone, resistance exercise, hindlimb suspension, recovery

## Abstract

**Purpose:** This study aimed to examine the exercise-mediated musculoskeletal recovery following hindlimb suspension (HS) in order to identify whether bone modeling and muscle hypertrophy would eventuate in a synchronized manner during recovery stage.

**Methods:** To identify whether 2-week HS would be sufficient to induce a significant reduction of physiological indices in both tibia and adjacent hindlimb muscles, a total of 20 rats was randomized into 2-week HS (*n* = 10) and age-matched control group (*n* = 10, CON). Another batch of rats were randomly assigned to three different groups to identify recovery intervention effects following suspension: (1) 2-week HS followed by 4-week spontaneous reloading recovery (HRE, *n* = 7). (2) 2-week HS followed by 4-week progressive resistance ladder climbing exercise (HEX, *n* = 7). (3) Age-matched control (CON, *n* = 7). DXA, micro-CT, and ^18^F-sodium fluoride (NaF) imaging, and EIA analysis were utilized to measure tibia bone indices. Hindlimb muscle wet weight and grip strength were measured to evaluate muscle mass and strength, respectively.

**Results:** In study 1, bone quality values [bone volume/total volume (BV/TV): -27%, areal bone mineral density (aBMD): -23%, mineral contents: -7.9%, mineral density: –4.1%, and bone density: -38.9%] and skeletal muscle weight (soleus: -46.8%, gastrocnemius: -19.6%, plantaris: -20.8%, TA: -22.8%, and EDL: -9.9%) were significantly lower in HS group compared to CON group. In study 2, micro-CT and DXA-based bone morphology (bone density, BT/TV, and aBMD) were fully recovered in HRE or HEX group. However, suspension-induced dysregulation of bone mineral metabolism was returned to age-matched control group in only HEX group, but not in HRE group. A greater level of biomarkers of bone formation (P1NF) and resorption (CTX-1) was observed in only HRE group compared to CON. The hindlimb skeletal muscle mass was significantly lower in both HRE and HEX groups compared to CON group. Hindlimb grip strength was the greatest in HEX group, followed by CON and HRE groups.

**Conclusion:** Following HS, progressive resistance exercise promotes recovery rates of bone and skeletal muscle strength without a significant increase in muscular mass, suggesting that exercise-induced reacquisition of bone and muscle strength is independent of muscle hypertrophy during early recovery stage.

## Introduction

Microstructure in bone tissue is constantly remodeled through the orchestrated actions of osteoclasts, osteoblasts, and osteocytes (Florencio-Silva et al., 2015). This dynamic process in conjunction with changes in muscle size is mainly modulated by repetitive mechanical resistance (Florencio-Silva et al., 2015). The musculoskeletal system is of critical importance in that it governs every movement of human body (Cointry et al., 2004). Since locomotion is mainly generated by bone’s ability that endure mechanical force induced by the contraction of adjacent skeletal muscles, it is considered that bone and skeletal muscle develop and decline in a similar fashion over the course of life (Cianferotti and Brandi, 2014). In line with this, accumulating evidence suggested that there are potential cellular interplays between the two organs (Brotto and Johnson, 2014). A promising theory has suggested that osteocyte senses muscle contraction-derived mechanical resistance and converts strains into biochemical signals, thus regulating bone modeling (Bonewald, 2011; Nakashima, 2015). Although molecular link that tunes bone and muscle seems to be evident (DiGirolamo et al., 2013), it is uncertain as to whether the recovery of both organs is in a complete synchronism during regeneration stage.

The adaptability of human bone has been well demonstrated in weightlessness environment. Microgravity during spaceflight induces bone loss at a rate of 0.5–1.5% a month (Lang et al., 2004). Such a loss tends to be persistent over 2.5 years following a stay on International Space Station (ISS) (Dana Carpenter et al., 2010; Leblanc et al., 2013). In addition to loss of bone quality, microgravity-induced muscle atrophy is evident. Astronauts subjected to a 17-day microgravity experienced an 8% decline in cross-sectional area of skeletal muscle (Tesch et al., 2005), thereby suggesting a reduction of bone and skeletal muscle under microgravity condition. Although human-based microgravity studies elicit direct clinical application, there is an extremely limited human-based data set due to the cost of performing large-scale experiment (Lloyd et al., 2014). A need for ground-based experiment of microgravity led to the development of a rodent hindlimb suspension (HS) model (Morey-Holton and Globus, 2002). Multiple studies demonstrated a potent reproducibility of HS-induced skeletal muscle atrophy and bone loss (Grano et al., 2002; Hurst and Fitts, 2003; Pan et al., 2008). Specifically, bone mass, density, mineralization, trabecular thickness, and osteoblastic activity are negatively affected following the chronic unloading treatment (Roer and Dillaman, 1990; Dehority et al., 1999; Bloomfield et al., 2002; Globus et al., 2015). In addition, bone loss with architectural disarray, tissue deterioration, and osteopenia are induced due to the suspension-induced absence of mechanical loading (Ohira et al., 2006).

Under spontaneous re-ambulation condition, experimental rodents fail to recover HS-induced loss of mineral content and bone formation rate during a period of recovery greater than the unloading duration, thereby suggesting that spontaneous normal weight bearing is not sufficient to induce fully recovered bone condition (Vico et al., 1995; Sakata et al., 1999). As compared with bone, suspension-induced reduction of muscle contractile properties has been documented to recover to pre-suspension level at a faster rate (Warren et al., 2004; Allen et al., 2006). The temporal difference of both organs’ recovery rate has been considered to induce a physiological mismatch in the production of force, thus increasing a possibility of musculoskeletal injury during rehabilitation (Allen et al., 2006). Several interventions have gained attention as countermeasures that preserve muscle and bone during or following a period of unloading (Ju et al., 2013; Jang et al., 2016). Among them, resistance exercise has been proven to be an effective strategy to accelerate the recovery rate of both organs. However, to the best of our knowledge, it has not been explored whether exercise-mediated recovery of both organs would occur in a complete simultaneity following HS.

Therefore, the purposes of this study were to investigate exercise and spontaneous reambulation-mediated recovery rate of tibia and related hindlimb muscles following HS. To achieve these aims, two related studies were performed. In study 1, 9-week-old SD rats were hindlimb-suspended for 2 weeks in order to confirm that 2-week HS would induce sufficient reduction of both muscle and bone. After the confirmation of our HS protocol, study 2 was performed by assigning second round of age-matched rats to each of the following three groups: (1) HS for 2 weeks followed by 4 weeks of spontaneous reloading recovery (HRE). (2) HS for 2 weeks followed by 4 weeks of progressive resistance ladder climbing exercise (HEX). (3) Another group of rats were housed in cages without HS during 6 weeks as an age-matched control (CON). We hypothesized that progressive resistance exercise would alter recovery rate of bone and/or muscle, as compared with weight bearing spontaneous recovery.

## Materials and Methods

### Animal Protocol

**FIGURE 1:**
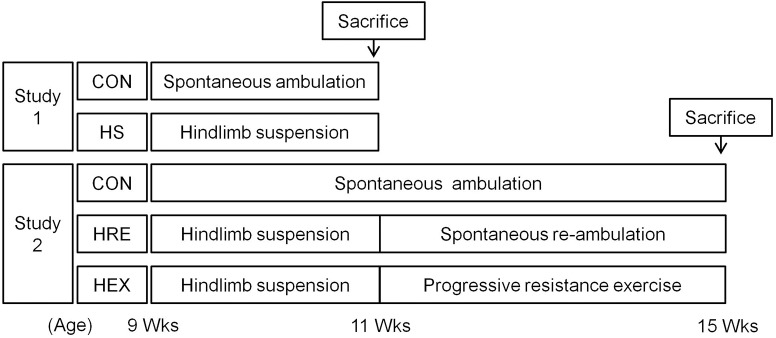
Schematic diagram of study design.

Sprague-Dawley rats (Orient Bio Co., Korea) were used in the study. All the rats were 9-week-old and were housed in a controlled environment in 12:12-h light–dark cycle at 22°C. The rats were fed with water and food (Rodent NIH-31 Open Formula Auto, Zeigler Bros Inc., United States) *ad libitum*. Experiments were approved by the Institutional Animal Care and Use Committee (IACUC) of Seoul National University. The IACUC number is SNU-150820-4-4. One week after arrival, first batch of rats were randomly assigned to either cage: control (CON, *n* = 10) or HS groups (*n* = 10) (**Figure 1**). In HS group, rats were taped on tails for HS for 2 weeks. Specifically, the rats were tail-suspended to the installed metal harness which holds rats in hindlimb suspended position by connecting taped tail to the pulley. Rats in control groups were housed in standard cages without access to metal harness for 2 weeks. They were used as an age-matched control against HS. After confirming whether 2-week HS protocol induced sufficient reduction of both bone and muscle, additional batch of rats were used to investigate the effects of spontaneous reloading and progressive resistance exercise on muscle and bone recovery following HS. Specifically, 1 week after arrival, another batch of rats were randomly assigned to three different groups: HS for 2 weeks followed by 4 weeks of spontaneous reloading recovery (HRE, *n* = 7), HS for 2 weeks followed by 4 weeks of progressive resistance ladder climbing exercise (HEX, *n* = 7). The rats in cages without any intervention were used as an age-matched control (CON, *n* = 7).

### Hindlimb Suspension Protocol

In order to make rat’s hindlimb to be suspended, taping was performed to attach rat tails to metal harness located above the ground surface. Two rats were housed in a HS cage (length – 60 cm × width – 30 cm × height – 40 cm) and were separated by an opaque barricade in order to eliminate any interaction between rats. Each rat was allowed to roam freely around in its own space (30 cm × 30 cm) by attaching their tail to a pulley allowing a 360° spin. The height of the taping was continuously adjusted according to a rat’s size in order for rats to stay in fully hindlimb suspended position. Grip strength was measured before and after intervention. The physical characteristics of the rats are presented in **Table 2**.

### Exercise Protocol

Progressive ladder climbing exercise was performed in HEX group following 2 weeks of HS treatment. The rats were subjected to a programmed exercise protocol 3 days a week based on a previous study (Westerlind et al., 1998). Exercise intervention was implemented on a 1-m ladder with 2-cm grid and 0.25-m width at 85° inclination against a wall. Since the rats’ physical function had been declined due to suspension treatment, exercise intensity was carefully managed in a progressive manner. Specifically, prior to the first exercise session, 1-day recovery period was provided to the rats. No weight was attached to rat’s tail at the start of exercise intervention. If the rats were unable to accomplish a certain weight load, previous weight load was used to encourage them to accomplish a total of 10 bouts of climbing. Only gentle brush to rats was used to encourage rats to accomplish each exercise bout. The detailed exercise protocol is presented in **Table 1**.

**Table 1:** Progressive resistance exercise protocol.

	Week 1	Week 2	Week 3	Week 4
Repetition	10 repetition		
% load/BW	0–50	60	70	80
Rest	2 min interval between repetition		
Frequency	3 sessions per week		

Progressive resistance ladder climbing exercise was performed in HEX group following 2 weeks of hindlimb suspension. Exercise intervention was implemented on a 1-m ladder with 2-cm grid and 0.25-m width at 85° inclination against a wall. Gentle brush to rats was used to encourage rats to accomplish each exercise bout. If the rats were unable to accomplish a certain weight load, previous weight load was used to encourage them to accomplish a total of 10 bouts.

### Bone Quality Analysis – PET/μCT and Micro-CT

*In vivo* Na^18^F PET/CT (positron emission tomography/computed tomography) scanning was performed to measure bone quality indices using a Nano PET/CT *in vivo* pre-clinical imager (Mediso, Hungary). Scanning was performed according to the protocol and procedure in the department of nuclear medicine in Seoul National University Bundang Hospital. Dynamic PET studies were performed after the intravenous application of ^18^F-fluoride. A dedicated PET-CT system with an axial field of view was operated in a three-dimensional mode and was used for all animals. The system provides the simultaneous acquisition of transverse slices with a slice thickness of 0.6 mm. The animals were positioned in the axial plane of the system to acquire the best resolution in the middle of the system. CT scan was performed prior to the PET scanning. Bone density was analyzed in Hounsfield Unit (HU) and trabecular BV/TV (bone volume/total volume) in percent (%). Each result was analyzed distinctively. All rats were anesthetized with isoflurane gas inhalation prior to scanning.

### Body Composition and Bone Mineral Density – DXA

*In vivo* DXA (Dual energy X-ray Absorptiometry) scanning was performed to measure body composition, whole body and areal bone mineral density (aBMD), and contents of rats using a Hologic Discovery W model (Hologic, United States). aBMD and areal bone mineral contents (aBMC) were taken from tibia of rats. For body composition indices, the values of fat mass, lean mass, and % fat were used for this study. All rats were anesthetized for DXA scanning and sacrifice was performed subsequently.

### Serum Bone Turnover Marker Analysis – EIA Analysis

The measurement of bone turnover markers in serum was performed by enzyme immunoassay (EIA) method. Procollagen of type I collagen (P1NP) was selected as a bone formation marker (Rat/Mouse P1NP EIA, Immunodiagnostic Systems). C-terminal telopeptide (CTX-1) was selected as a bone resorption marker (RatLaps^TM^ CTX-1 EIA, Immunodiagnostic Systems). The sensitivity of P1NP in the analysis was 0.7 ng/mL, and the inter-assay and intra-assay CV were 9.2–8.2 and 6.4–5.0%. The CTX-1 RatLaps^TM^ detection limit was 2.0 ng/mL, and the inter-assay and intra-assay coefficients of variation were 9.2–5.8 and 14.8–10.7%, respectively.

### Grip Strength

The grip strength was measured using Grip Strength Meter (Bioseb, France). The test was performed in rat’s hindlimb by allowing the animals to grasp a bar attached to the force gauge, followed by pulling the animal away from the gauge in a progressive manner. Specifically, rats were positioned by allowing it to put its forelimb onto the researcher’s left hand to avoid any interference of its forelimb to the gauge. The test was performed five times in each hindlimb.

### Skeletal Muscle Collection

For skeletal muscle dissection, an incision was made through the skin around ankle area and the skin was reflected to expose the muscles of the lower leg. The Achilles tendon was cut to remove soleus, plantaris, and gastrocnemius. To dissect tibialis anterior and extensor distorum longus, the fascia covering the muscles was carefully removed and extensor ligament was cut to release the distal tendon and use it to peel them off with a fine surgical scissors. The dissected muscles were cleaned of excess fat and external connective tissue before the measurement.

### Statistical Analysis

Statistical analysis was performed using SPSS 22.0 software. Results were expressed as mean ± SEM. *T*-test was performed to examine the difference between two groups in study 1. One-way ANOVA was performed to examine the difference in study 2, followed by Bonferroni’s post hoc test. The level of significance was set at *p* < 0.05.

## Results

### The Effects of a 2-Week Hindlimb Suspension on Body Composition

Hindlimb suspension of experimental rats for 14 days resulted in significant changes in body composition (**Table 2**). Total mass (–11.3%) and lean mass (–15.2%) were significantly decreased after 14 days of HS compared to age-matched controls. Although total fat mass was not changed statistically, body fat (38.1%) was significantly increased by HS.

**Table 2:** Physical characteristics after hindlimb suspension.

	CON (*n* = 10)	Hindlimb suspension (*n* = 10)
Total mass (g)	335.250 ± 3.918	301.690 ± 7.339^∗∗^
Lean mass (g)	288.190 ± 4.071	250.330 ± 6.137^∗∗∗^
Fat mass (g)	36.290 ± 2.425	41.420 ± 1.754
Fat (%)	10.840 ± 0.718	13.800 ± 0.453^∗∗^

Lean mass, fat mass, and percent fat were measured by DXA analysis following 2-week hindlimb suspension (*n* = 10). Age-matched rats were used as control (*n* = 10). All the results were presented as mean ± SEM, ^∗∗^*p* < 0.01 vs. CON, ^∗∗∗^*p* < 0.001 vs. CON.

### The Effects of a 2-Week Hindlimb Suspension on Bone and Muscle

**FIGURE 2:**
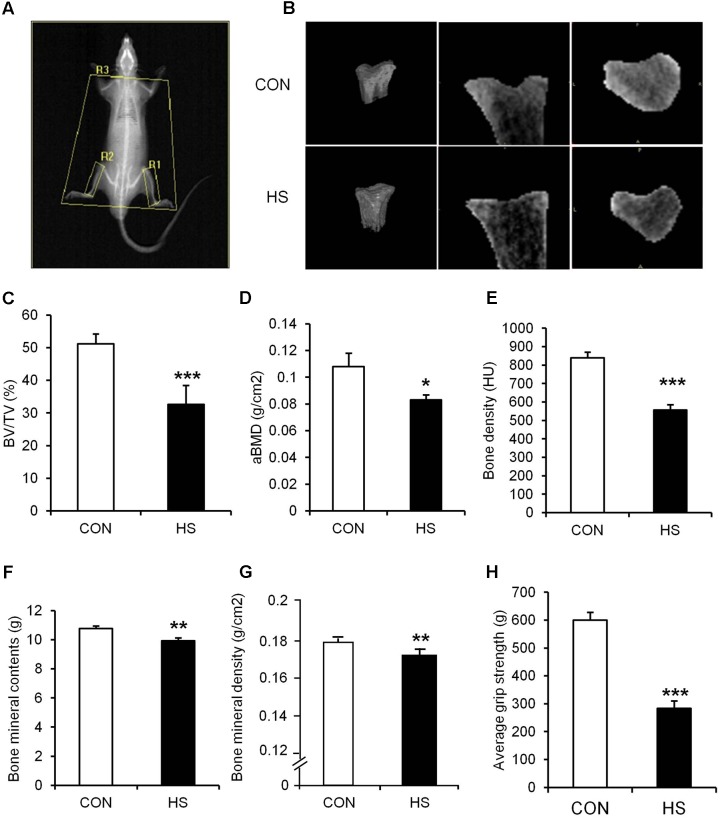
The effects of hindlimb suspension on bone morphology and grip strength. **(A)** Image of ROI area selection of DXA scan (R1, right tibia; R2, left tibia; R3, whole body without head/global: whole body). **(B)** Representative micro-CT images of animals’ tibiae. **(C)** BV/TV, **(D)** aBMD, **(E)** bone density, **(F)** bone mineral contents, **(G)** bone mineral density, and **(H)** hindlimb grip strength were measured after 2-week hindlimb suspension. ^∗^*p* < 0.05 vs. CON, ^∗∗^*p* < 0.01 vs. CON, ^∗∗∗^*p* < 0.001 vs. CON. All the results were presented as mean ± SE.

Hindlimb suspension led to significant changes in tibia and four different hindlimb muscles (**Figure 2** and **Table 3**). Specifically, tibia bone density (–33.8%), BT/AT (–37.3%), aBMD (–33.2%), bone mineral contents (–7.5%), and bone mineral density (–4.1%) were decreased significantly after HS. Serum P1NP (biomarker of bone formation) levels were significantly lower in HS group compared to CON group (*p* < 0.009). Serum CTX-1 (biomarker of bone resorption) level was not different between two groups. The wet weight of four different skeletal muscles adjacent to tibia was measured during surgical procedure. Soleus (–46.8%), gastrocnemius (–19.6%), tibialis anterior (–12.1%), extensor distorum longus (–9.9%), and plantaris (–19.8%) were decreased significantly in HS group compared to age-matched control.

**Table 3:** Skeletal muscles weight following hindlimb suspension.

	CON (*n* = 10)	Hindlimb suspension (*n* = 10)
Soleus (g)	0.265 ± 0.011	0.141 ± 0.007^∗∗∗^
Gastrocnemius (g)	3.383 ± 0.054	2.722 ± 0.071^∗∗∗^
TA (g)	1.242 ± 0.018	1.092 ± 0.033^∗∗^
EDL (g)	0.3178 ± 0.0116	0.2863 ± 0.0061^∗^
Plantaris (g)	0.6799 ± 0.0164	0.5385 ± 0.0179^∗^

*Skeletal muscles adjacent to tibia were weighted following 2-week hindlimb suspension *n* = 10). Age-matched rats were used as control (*n* = 10). TA, tibialis anterior; EDL, extensor digitorum longus. All the results were presented as mean ± SEM, ^∗^*p* < 0.05 vs. CON, ^∗∗^*p* < 0.01 vs. CON, ^∗∗∗^*p* < 0.001 vs. CON.*

### Spontaneous Ambulation and Resistance Exercise-Mediated Bone Turnover Rate Following Hindlimb Suspension

**FIGURE 3:**
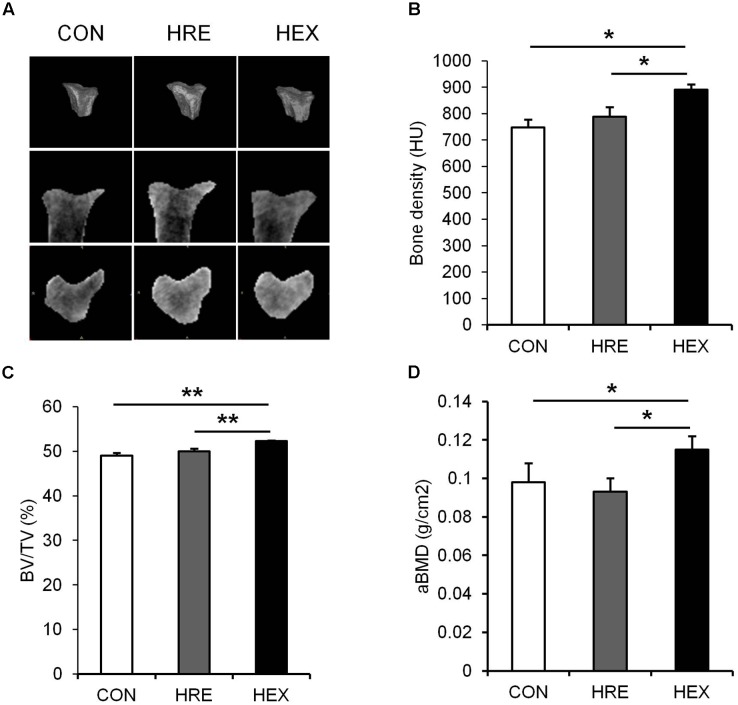
The effects of re-ambulation and exercise on bone morphology following hindlimb suspension. **(A)** Representative micro-CT images of animals’ tibiae. **(B)** Bone density, **(C)** BV/TV, and **(D)** aBMD were measured after 4-week intervention. ^∗^*p* < 0.05, ^∗∗^*p* < 0.01. All the results were presented as mean ± SE.

The Na^18^F PET/CT scan was performed and the lower portion of rats were focused for the tibia metaphysis analysis (**Figure 3**). The result shows that mineral metabolism (bone turnover rate) of both sides of tibia was significantly higher in HRE group compared to CON and HEX groups (*p* < 0.05). Serum levels of P1NP (biomarker of bone formation) and CTX-1 (biomarker of bone resorption) were also significantly higher in HRE group compared to CON and HEX groups (*p* < 0.05).

### Spontaneous Ambulation and Resistance Exercise-Mediated Bone Qualities Following Hindlimb Suspension

**FIGURE 4:**
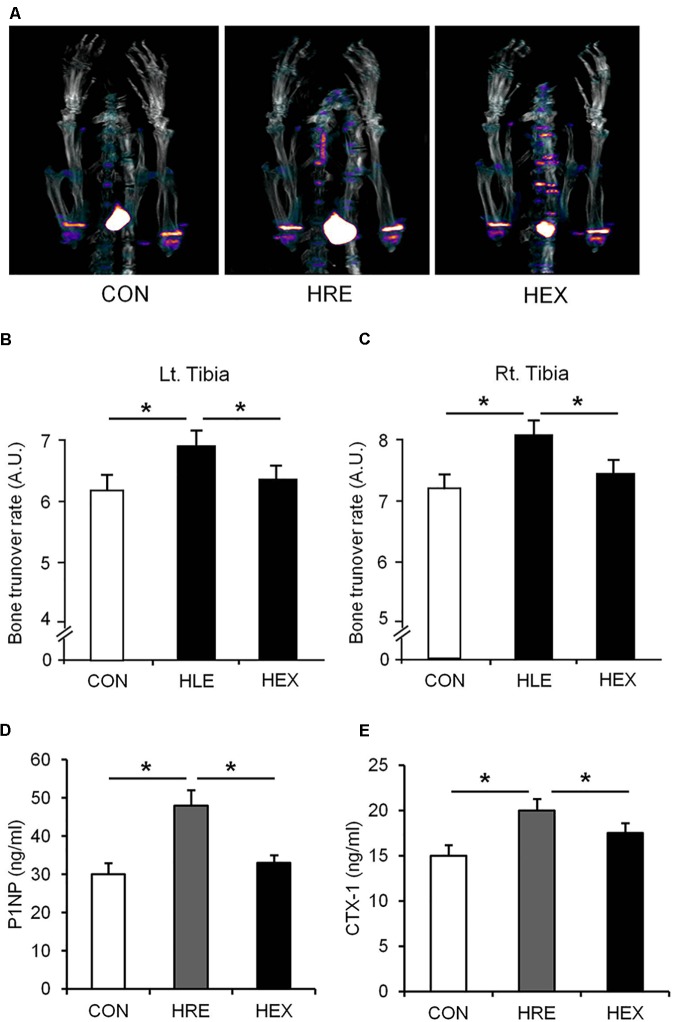
The effects of re-ambulation and exercise on bone mineral metabolism following hindlimb suspension. **(A)** Representative PET/CT fusion images of tibia metaphysis. Bone turnover rate of **(B)** left and **(C)** right tibia was obtained from PET/CT fusion images. Biomarkers of **(D)** bone formation (P1NP) and **(E)** bone resorption (CTX-1) were measured by EIA analysis. ^∗^*p* < 0.05. All the results were presented as mean ± SE.

Tibia-specific regional micro-CT scanning was performed to obtain three-dimensional and cross sectional images of tibia for qualitative analysis (**Figure 4**). Bone density was analyzed in HU and trabecular BV/TV in percent. Bone density (*p* < 0.05), BV/TV (*p* < 0.01), and aBMD (*p* < 0.05) were significantly higher in HEX group compared to CON and HRE groups. There was no difference observed between CON and HRE groups.

### Spontaneous Ambulation and Resistance Exercise-Mediated Muscle Weight Following Hindlimb Suspension

Four different skeletal muscles adjacent to tibia were weighed during surgical procedure (**Table 4**). The weights of soleus (*p* < 0.01), gastrocnemius, tibialis anterior, EDL, and plantaris (*p* < 0.05) were significantly lower in HRE and HEX groups compared to age-matched control. However, these muscles’ weight was not statistically different between HRE and HEX groups.

**Table 4:** Recovery intervention-mediated skeletal muscles weight following hindlimb suspension.

	CON (*n* = 7)	HRE (*n* = 7)	HEX (*n* = 7)
Soleus (g)	0.4021 ± 0.0181	0.3692 ± 0.0129^∗∗^	0.3539 ± 0.0093^∗∗^
Gastrocnemius (g)	4.9505 ± 0.1365	4.6156 ± 0.0733^∗^	4.5153 ± 0.0741^∗^
Tibialis anterior (g)	1.9068 ± 0.0615	1.7592 ± 0.0363^∗^	1.6978 ± 0.0482^∗^
EDL (g)	0.4643 ± 0.0246	0.4297 ± 0.0078^∗^	0.4224 ± 0.0054^∗^
Plantaris (g)	0.9712 ± 0.0348	0.9396 ± 0.0179^∗^	0.9271 ± 0.0196^∗^

Skeletal muscles adjacent to tibia were weighted following 4-week recovery intervention (*n* = 7). Age-matched rats were used as control (*n* = 7). TA, tibialis anterior; EDL, extensor digitorum longus. All the results were presented as mean ± SEM, ^∗^*p* < 0.05 vs. CON, ^∗∗^*p* < 0.01 vs. CON.

### Hindlimb Grip Strength Before and After Spontaneous Ambulation and Resistance Exercise

**FIGURE 5:**
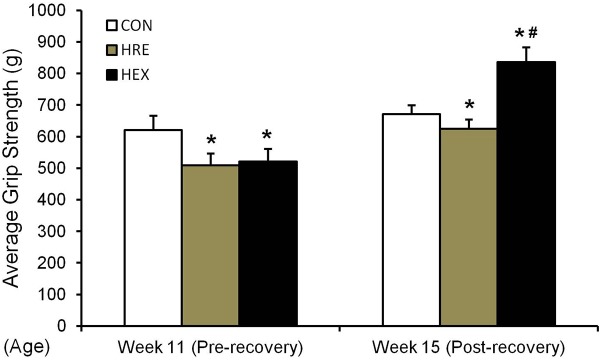
Hindlimb grip strength before and after recovery intervention. The grip strength test was performed in rat’s hindlimb by allowing the animals to grasp a bar attached to the force gauge, followed by pulling the animal away from the gauge in a progressive manner. The test was performed five times. ^∗^*p* < 0.05 vs. CON, ^#^*p* < 0.05 vs. HRE. All the results were presented as mean + SE.

The grip strength was calculated by the average of five records of each rat. In study 1 (**Figure 2H**), grip strength was significantly lower in HS group compared to CON (*p* < 0.05). In study 2 (**Figure 5**), grip strength was lower in HRE and HEX groups compared to age-matched control before recovery intervention (*p* < 0.05). Following recovery intervention, grip strength was the highest in HEX group, followed by CON and HRE (*p* < 0.05).

## Discussion

The main purpose of this study was to investigate progressive resistance exercise-mediated recovery indices of bone and skeletal muscles following 2-week HS. Our results clearly showed that 2-week HS induces a significant reduction of physiological indices in both tibia and adjacent hindlimb muscles. Interestingly, progressive resistance exercise promoted recovery rates of tibia and hindlimb muscular strength in the absence of an increase of related muscle mass.

### The Effects of Hindlimb Suspension on Bone and Muscles

Hindlimb suspension is a well-documented model as ground-based analog to identify the effects of unloading condition on musculoskeletal system, thereby resulting in concomitant loss of both muscle and bone (Morey-Holton et al., 2005; Colaianni et al., 2017). In accordance with previous findings (Swift et al., 2010; Jing et al., 2014), our results revealed significant deterioration of both tissues after only 2 weeks of suspension (**Figure 2** and **Table 3**), validating our HS protocol. In the study, the weights of five major skeletal muscles (soleus, gastrocnemius, tibialis anterior, EDL, and plantaris) were measured due to their anatomical proximity to tibia. Interestingly, the reduction of individual muscle weight was determined to be in a broad spectrum (soleus: -46.8%, gastrocnemius: -19.6%, tibialis anterior: -12.1%, EDL: -9.9%, and plantaris: -19.8%). This implies a possibility that microgravity-simulated atrophy is induced in a muscle type-dependent manner. This idea is further supported by several lines of studies indicating that the loss of protein was more dramatic in slow-twitch muscles than fast-twitch muscles (Tsika et al., 1987; Hornberger et al., 2001; Wang et al., 2017). Based on the fact that soleus muscle presents a predominance of slow twitch type I fibers while the other four muscles consist of mainly fast twitch type II fibers or a mixture (Cornachione et al., 2011), it is considered that the different atrophy rates are attributed to the different fiber type composition in each muscle.

The muscle atrophies concomitant with the suspension-mediated bone loss (bone density, BV/TV, aBMD, bone mineral contents, and bone mineral density) indicates that 2-week hindlimb suspension is sufficient to induce a significant deterioration of musculoskeletal system.

### The Effects of Spontaneous Reambulation and Resistance Exercise on the Recovery of Bone and Muscles

A variety of approaches have been implemented to counteract the loss of bone and skeletal muscles. Although exercise is considered as an efficient and practical strategy to facilitate the recovery of both tissues, most of the related studies has been focused on either muscle or bone (Ju et al., 2013; Song et al., 2017), limiting a comprehensive understanding of physiological link between two tissues during recovery stage.

As our multi-faceted approaches demonstrated, a greater recovery of micro-CT (bone density and BV/TV) and DXA-based bone parameters (aBMD) was observed in HEX group compared to HRE group (**Figure 3**). These results indicate that morphological recovery of the bones was promoted by progressive resistance exercise, which are in line with a previous study indicating the therapeutic effect of exercise on bone (Shirazi-Fard et al., 2014). Bone is a dynamic tissue changing its strength and micro-morphologic features by regulating bone turnover rate *via* actions of osteoblasts and osteoclasts (van der Eerden et al., 2014). Weakness of bone strength and stiffness is the manifestation of excessive or deficient bone turnover rate. ^18^F-sodium fluoride (NaF) imaging has been proven to catch calcium metabolic activity in the bone structures, which could be easily unnoticed from analyzing the morphological images such as micro-CT and DXA (Jadvar et al., 2015). Thus, bone turnover rate (bone mineral metabolism) was measured using ^18^F-NaF for further investigation. Interestingly, despite of complete recovery of the CT- and DXA-based bone morphology in HLE group compared to age-matched control, tibia mineral metabolism was abnormally elevated in HRE group compared to CON and HEX group (**Figure 4**), providing a piece of biochemical evidence that post-suspension bone modeling was not completed under the condition of spontaneous reambulation. In order to intensify the interpretation of the dysregulated bone turnover rate, serum P1NP (bone formation) and CTX-1 (bone resorption) were tested as surrogate markers of osteoblast and osteoclast activities. In agreement of our PET/CT data, these markers were significantly elevated in HRE group compared to both CON and HEX group (**Figures 4D,E**). Considering our experimental design of 2-week HS and subsequent 4-week recovery intervention, it is considered that tibia mineral metabolism requires at least more than two times the duration of reambulation for the complete recovery. This idea is in agreement with a previous study that bone requires nearly three times the period of re-ambulation to return to age-matched control levels (Allen et al., 2006). In addition, with multi-faceted analyses of DXA, micro-CT, and ^18^F-NaF imaging, the current study supports a notion that the duration of morphological and biochemical bone recovery could be significantly reduced by progressive resistance exercise (Russo, 2009; Shirazi-Fard et al., 2014).

In the study, five major muscles adjacent to tibia were weighed to exam whether progressive resistance exercise would induce the re-acquisition of muscle mass concomitant with improved bone qualities. Interestingly, all the muscles’ weight was not different between HRE and HEX groups while the grip strength was the greatest in HEX group (**Table 4** and **Figure 5**). The increased force production without regain of muscle mass implies a possibility of peripheral neural recovery-mediated force production under early regeneration phases. This idea is supported by a recent study on peripheral motor axons, indicating that suspension-induced reduction of a neuromuscular axonal excitability property returns to control range after a short duration of recovery (Banzrai et al., 2016). Thus, it is considered that progressive resistance exercise induces a faster recovery of peripheral nervous system compared to muscle hypertrophy to fine-tune force generation in order to prevent skeletal muscle dominance-induced fracture under the regeneration process.

## Conclusion

Our multi-faceted study demonstrated that progressive resistance exercise following HS promotes recovery rates of bone morphology and skeletal muscle strength in the absence of a significant increase in muscle mass. This suggests that exercise-induced regain of muscular and bone strength is independent of muscle hypertrophy during early recovery stage. In future endeavor, a greater understanding of physiological link among muscle, bone, and neuroaxonal properties would provide additional insight into the development of promising strategies to counteract musculoskeletal and neuromuscular pathophysiological condition.

## Author Contributions

All authors listed have made a substantial and intellectual contribution to the work, and approved it for publication. HS contributed in the acquisition and analysis of data, and drafting the work. SC and H-YL contributed in the acquisition and analysis of data for the work. HL contributed in the interpretation of data for the work, revising the work critically, and final approval of the version to be published. WS contributed in the conception of the work, revising the work critically, and final approval of the version to be published.

## Conflict of Interest Statement

The authors declare that the research was conducted in the absence of any commercial or financial relationships that could be construed as a potential conflict of interest.
